# Bilateral ultrasound-guided maxillary and mandibular combined nerves block reduces morphine consumption after double-jaw orthognathic surgery: a randomized controlled trial

**DOI:** 10.1136/rapm-2024-105497

**Published:** 2024-05-02

**Authors:** Thomas Esquerré, Marion Mure, Vincent Minville, Alice Prevost, Frédéric Lauwers, Fabrice Ferré

**Affiliations:** 1 Department of Anesthesiology, Intensive Care and Perioperative Medicine, University Hospital Centre Toulouse, Toulouse, France; 2 Department of Plastic and Maxillofacial Surgery, University Hospital Centre Toulouse, Toulouse, France

**Keywords:** REGIONAL ANESTHESIA, Nerve Block, Pain, Postoperative, Ultrasonography

## Abstract

**Background:**

Double-jaw surgeries are known to be painful and to require opioids. Maxillary (V2) and mandibular (V3) nerves block could provide adequate pain management with minimal opioid-related side effects. Our main objective was to evaluate the analgesic effect of bilateral ultrasound-guided V2 and V3 combined nerves block in patients undergoing double-jaw orthognathic surgery.

**Methods:**

In this single-blind, randomized control study, 50 patients were prospectively allocated to either bilateral ultrasound-guided V2 and V3 combined nerves block or intraoral infiltration of local anesthetic. Primary outcome was the cumulative oral morphine equivalent (OME) consumption assessed at postoperative day 1. Secondary outcomes were cumulative OME consumption and pain scores in recovery room and at postoperative day 2, intraoperative anesthetic consumption, and opioid-related side effects. Preoperative anxiety was investigated by the Amsterdam Preoperative Anxiety and Information Scale (APAIS).

**Results:**

Compared with infiltration, ultrasound-guided regional anesthesia reduced cumulative OME consumption on day 1 (45.7±37.6 mg vs 25.5±19.8 mg, respectively, mean difference of −20.1 (95% CI −37.4 to −2.9) mg, p=0.023) and day 2 (64.5±60 mg vs 35.8±30.2 mg, respectively, mean difference of −28.7 (95% CI −55.9 to −1.43) mg, p=0.040). Interestingly, worst pain score and cumulative OME consumptions on day 2 were positively correlated with the APAIS (Pearson’s correlation coefficient of 0.42 (p=0.003) and 0.39 (p=0.006), respectively).

**Conclusion:**

Bilateral ultrasound-guided V2 and V3 combined nerves block reduces postoperative opioid consumption by about 50% in patients undergoing double-jaw surgery.

**Trial registration number:**

NCT05351151.

**WHAT IS ALREADY KNOWN ON THIS TOPIC**

- Literature suggests analgesic effect of maxillary (V2) or mandibular (V3) nerve blocks in maxillofacial surgery.

**WHAT THIS STUDY ADDS**

- This randomized controlled trial clearly demonstrated that bilateral ultrasound-guided V2 and V3 combined nerves block is an efficient regional anesthesia which provides a clinically significant opioid-sparing effect in the first 24 hours after double-jaw orthognathic surgery.

**HOW THIS STUDY MIGHT AFFECT RESEARCH, PRACTICE OR POLICY**

- Ultrasound-guided regional anesthesia of V2 and V3 nerves could be efficient for most of maxillofacial surgeries.

## Introduction

 Double-jaw orthognathic osteotomy is a common maxillofacial surgery allowing the correction of dental joint or facial esthetic disorders, as well as sleep apnea syndrome.^1^ It consists in double osteotomy of maxillary and mandibular bones mainly involving maxillary (V2) and mandibular (V3) nerves and is associated with intense postoperative pain requiring opioids.^2^

By providing adequate pain management with minimal opioid-related side effects, regional anesthesia (RA) might facilitate rehabilitation and also reduce the likelihood of complications but, to date, there is no recommendation from the French Anesthesia and Intensive Care Society for RA in maxillofacial surgeries.^3^ Hence, the postoperative pain management of patients undergoing double-jaw surgery is usually based on local infiltration analgesia of mucosa (ie, intraoperative injection of local anesthetics) combined with a multimodal systemic analgesia regimen including opioids.^4^,^6^

Previous study demonstrated the analgesic benefits of an ultrasound-guided V2 nerve block with 50% reduction in opioid consumption on postoperative day 2 in pediatric patients undergoing cleft palate surgeries.^7^ More recently, encouraging results were published suggesting the analgesic effectiveness of this nerve block for orthognathic surgery in adults but with a low level of evidence.^8^,^10^

Several case series studies have emphasized the analgesic effect of inferior alveolar nerve (IAN) block in cancer or orthognathic mandibular surgeries, but results are conflicting.^11^,^14^ However, it is suggested that orthognathic surgery generates pain in territories that are not only innervated by IAN.^11^,^13^ To go further, an ultrasound-guided V3 nerve block performed immediately after the foramen ovale and just before its first ramifications could improve pain relief and morphine consumption in mandibular osteotomies and fracture surgeries.^15 16^

To the best of our knowledge, only two studies have investigated the combination of V2 and V3 (or IAN) nerves block for maxillomandibular surgeries. They suggest that RA could reduce postoperative pain and optimize intraoperative conditions.^17 18^

The aim of our study was to evaluate the analgesic effect of a combined, bilateral, ultrasound-guided V2 and V3 nerves block in patients undergoing scheduled double-jaw orthognathic surgery. We made the assumption that this RA could reduce cumulative opioid consumption in the first postoperative days.

## Materials and methods

### Design of the study

This interventional, single-blind randomized controlled trial was conducted at the University Teaching Hospital of Toulouse, France, from May 2022 to July 2023. Written informed consent was obtained for all subjects. This trial was registered before patient enrollment on ClinicalTrials.gov (NCT05351151; principal investigator: MM; registered on 22 April 2022).

### Inclusion and exclusion criteria

All patients aged 15–45 years scheduled for elective primary double-jaw surgery under general anesthesia were eligible. The exclusion criteria were: American Society of Anesthesiologists score ≥3; preoperative use of opioids; pre-existing neurological deficit in maxillary or mandibular area; patient refusal; major spontaneous or acquired hemostasis disorders; infection at the puncture site; allergy to local anesthetics; and pregnancy/breast feeding; inclusion in other clinical study; guardianship, people with cognitive trouble.

### Randomization

Using computer-generated randomization (Stata Statistical Software, Release 14, StataCorp, College Station, Texas, USA) with block size of 4, patients were successively randomly assigned to either bilateral ultrasound-guided V2 and V3 combined nerves block (RA group) or intraoral infiltration of local anesthetics (infiltration group). Allocation numbers were sealed in envelopes and opened successively at the time of inclusion on the day of surgery by the anesthesiologist in charge of the patient and who performed the overall anesthetic management. Patients were blinded to their group assignment (RA and infiltration were performed under general anesthesia). The physician who evaluated the outcome criteria was blinded after assignment to interventions.

### General anesthesia

A standardized anesthetic protocol was applied to all allocated patients of the study. Induction as well as maintenance of anesthesia was carried out by propofol and remifentanil target-controlled infusion (Primea Base, Minto Model—brain target between 3 and 6 ng/mL). The level of intraoperative sedation and nociception were monitored using the bispectral index (BIS; Medtronic, Brampton, Canada, target between 40–60) and the analgesia nociception index (ANI; MDoloris Medical Systems, Lille, France, target >50). All patients received dexamethasone 8 mg intravenous and ketamine intravenous (initial bolus of 0.5 mg/kg, and then 0.15 mg/kg/h stopped 30 min before the end of surgery). All patients received 1 g of tranexamic acid before incision and antibiotic prophylaxis according to our guidelines. Blood pressure was controlled with urapidil infusion in case of arterial hypertension for a mean arterial pressure targeted between 60 and 70 mm Hg once confounding factors excluded, for example, lack of analgesia (ANI<50) and/or sedation (BIS>60). Droperidol 0.625 mg intravenous was injected before wake-up to prevent postoperative nausea and/or vomiting (PONV) only for patients with Apfel score ≥3.

### Surgical infiltration

For the patients allocated to the RA group, intraoral surgical infiltration of mucosa was carried out with epinephrine-infused isotonic saline at 0.005 mg/mL (maximum volume of 20 mL). For the patients allocated to the infiltration group, intraoral surgical infiltration of mucosa was carried out with lidocaine 10 mg/mL with added epinephrine 0.005 mg/mL (maximum volume of 20 mL).

### Ultrasound-guided V2 and V3 nerves block

**Figure 1:**
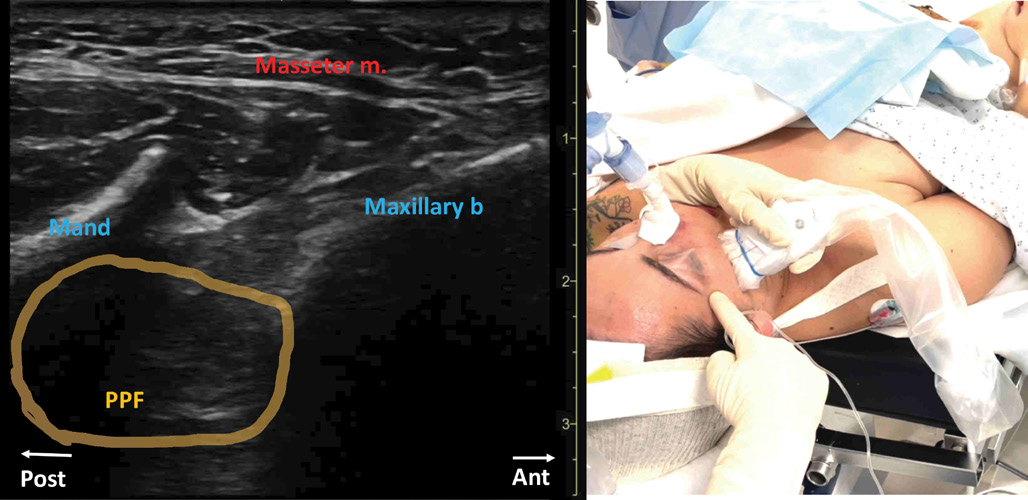
Out-of-plane suprazygomatic right V2 nerve block. Puncture point is pointed by the index finger. Ultrasound probe in place. Mand, ascending process of the mandible; Masseter m, masseter muscle; Maxillary b, maxillary bone; PPF, pterygopalatine fossa.

For patients allocated to the RA group, bilateral ultrasound-guided V2 and V3 combined nerves block was performed under general anesthesia and before starting the surgery. Illustrative videos are available in the online supplemental digital content (V2blocknerve_SDC1.mp4 and V3blocknerve_SDC2.mp4 for respectively V2 and V3 block nerves). V2 nerve block was performed via a suprazygomatic approach.^7 19 20^ Briefly, the needle (B Braun Stimuplex Ultra 360, 50 mm—22 G) was inserted toward the pterygopalatine fossa (HFL50× 15-6 MHz, S-Nerve II, Fujifilm Sonosite, Bothell, Washington, USA), out of the plane of the ultrasound (figure 1). An aspiration test has to be negative prior to any injection. Then, 5 mL of ropivacaine 4.75 mg/mL was slowly injected.

**Figure 2:**
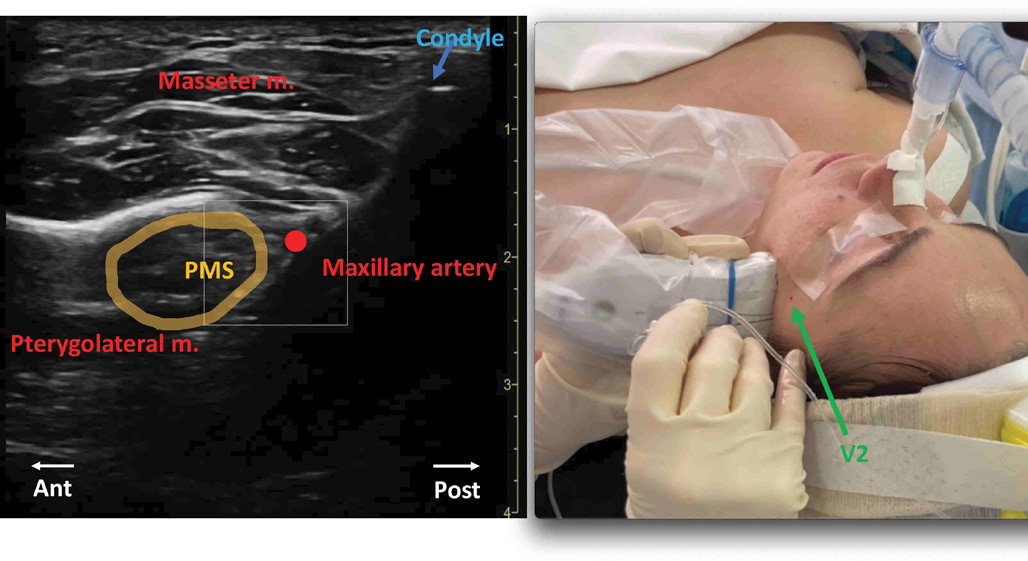
Out-of-plane right V3 nerve block. Puncture point (V3) at the center’s mark of the ultrasound probe, corresponding at the mandibular notch. Puncture point of the maxillary nerve block (V2) procedure is visible (green arrow). Masseter m, masseter muscle; PMS, pterygomandibular space; Pterygolateral m, pterygolateral muscle.

Ultrasound-guided V3 nerve block was performed as previously described.^16 20^ The probe was positioned more posteriorly compared with the V2 nerve block (HFL50× 15-6 MHz, S-Nerve II, Fujifilm Sonosite). Pterygomandibular space was first identified between coronoid process and condyle with the patient’s mouth open (figure 2). The needle (B Braun Stimuplex Ultra 360, 50 mm—22 G) was inserted out of the plane of the ultrasound through an infrazygomatic route close to maxillary artery. After aspiration test, 5 mL of ropivacaine 4.75 mg/mL was slowly injected.

### Surgery

Surgical procedure was standardized, therefore performed in the same way by all surgeons. Maxillary osteotomy consisted of a LeFort I osteotomy, and the mandibular osteotomy consisted of a bilateral sagittal split osteotomy according to Epker.^21 22^ Osteotomies were carried out with a piezoelectric scalpel. Closure was performed without drainage system.

### Postoperative analgesia

Postoperative analgesic management was standardized. At the end of surgery, patients received 1 g intravenous paracetamol, 100 mg intravenous ketoprofen and 20 mg intravenous nefopam in the absence of any contraindication. In the postanesthesia care unit (PACU), pain was evaluated by an 11-point numerical rating scale (NRS) where 0 is no pain and 10 is the worst pain imaginable. Intravenous morphine was administered when the NRS was >3, with an initial bolus of 0.05 mg/kg, and then 2 mg every 5 min until NRS≤3. After discharge from the PACU, all patients received oral paracetamol 1 g every 6 hours for 7 days, oral ibuprofen 400 mg every 8 hours for 5 days, and oral morphine 10 mg every 4 hours, if necessary (ie, NRS>3), for 5 days.

### Endpoints

Patients were followed up for 2 days after surgery. The primary endpoint was the total morphine consumption in the first 24 hours after surgery (ie, at day 1) compared between groups. Consumption was expressed as oral morphine equivalent (OME) and calculated as follows: 1 mg intravenous morphine=3 mg OME. The second outcomes were: postoperative cumulative opioid consumptions in the PACU and at 48 hours after surgery (ie, at day 2), worst postoperative pain score (NRS) in PACU, day 1 and day 2 recorded by structured interview, intraoperative blood loss, anesthetic reagents consumption, anesthetic and surgical durations and side effect monitoring. Correlations between preoperative anxiety (Amsterdam Preoperative Anxiety and Information Scale, APAIS) and postoperative pain/OME consumption were assessed at day 2.

### Sample size calculation

Data from the literature suggest that patients consume about 45–75 mg OME at postoperative day 1. It is worth noting that no intraoperative surgical infiltration was achieved in these previously published studies.^2 4^ Furthermore, a pilot study conducted in our department including 50 patients who benefit from V2 and V3 combined nerves block in the setting of an adequate postoperative multimodal analgesia regimen revealed an OME consumption of 18 mg (±15 mg) at day 1. Our working hypothesis was that RA would reduce morphine consumption by ≥50% compared with infiltration (difference of means between groups=20 mg). To demonstrate a significant difference in analgesic consumption between the RA and infiltration groups with an alpha risk set at 5% and a power set at 90%, 22 patients per group had to be analyzed. In order to compensate for possible inclusion errors and patients lost to follow-up (generally estimated at 10%), 25 patients were planned to be included in each group.

### Statistical analysis

The normality of the data was verified using the Shapiro-Wilk test. Quantitative variables were expressed as median (25th–75th percentiles) or mean±SD as appropriate. Categorical variables were expressed as numbers (%). The comparison of continuous variables between the RA and infiltration groups was performed using the Student’s t-test or Mann-Whitney U test as appropriate. Categorical variables were compared using the χ^2^ or Fisher’s exact test. The time course of pain and morphine consumption were studied using a repeated measures analysis of variance (ANOVA). Two factors and their interaction were studied: the group effect (ie, RA vs infiltration) and the time effect (PACU, day 1, day 2). The correlation between preoperative anxiety (APAIS) and OME consumption/worst pain score at day 2 was evaluated by Pearson’s correlation. Statistical analysis was performed using MedCalc software (V.12.6.1, MedCalc Software, Ostend, Belgium; 2013). A p value ≤0.05 was considered statistically significant.

## Results

**Figure 3:**
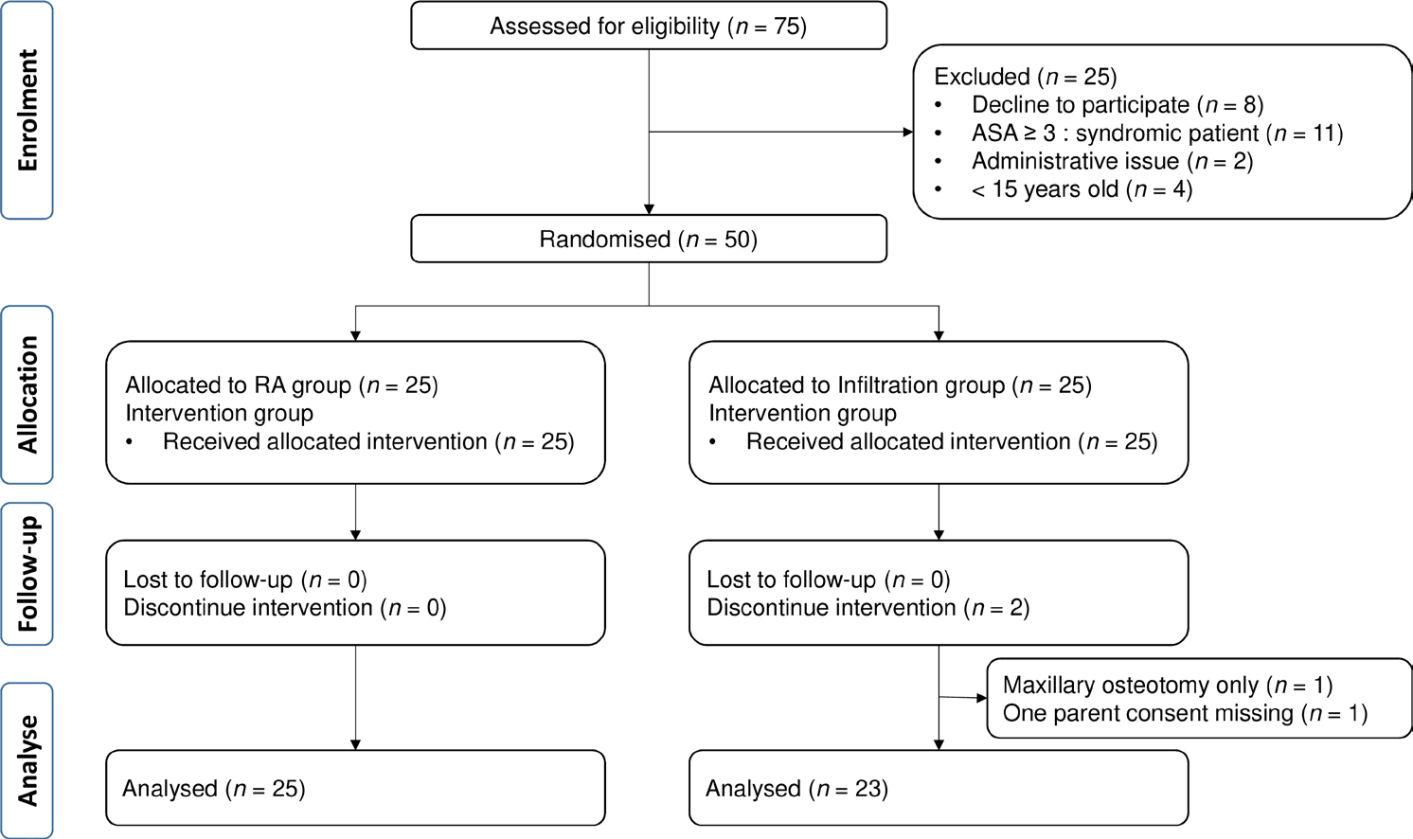
Consolidated Standards of Reporting Trials (CONSORT) flow diagram. ASA, American Society of Anesthesiologists; RA, regional anesthesia.

From May 2022 to July 2023, seventy-five patients scheduled for a maxillomandibular osteotomy surgery were eligible in our unit. 50 patients (65.7%) were finally consecutively included in our trial and 48 completed the study (figure 3).

Demographic characteristics, including preoperative APAIS, were comparable between groups (table 1).

**Table 1:** Baseline characteristics

	RA group n=25	Infiltration group n=23
Median age, years	19 (17–33)	23 (18–32)
Sex, n (%)		
Male	11 (44)	11 (48)
Female	24 (56)	22 (52)
Median weight, kg	60 (52.5–71.5)	67 (55.5–74.5)
Median BMI, kg/m²	20.8 (19.1–23.5)	21.7 (20.2–24.8)
Global APAIS score	15.2±4.6	16.9±6.3
After surgery, n (%)		
Outpatient	17 (68)	14 (61)
Surgery ward	8 (32)	9 (39)

Data are expressed as n (%), mean±SD or median (25th–75th percentiles) as appropriate.

No statistical difference was found between groups.

APAIS, Amsterdam Preoperative Anxiety and Information Scale; BMI, body mass index; RA, regional anesthesia.

Compared with infiltration, RA reduced cumulative OME consumption on day 1 (45.7±37.6 mg vs 25.5±19.8 mg, respectively, mean difference of −20.1 (95% CI −37.4 to −2.9) mg, p=0.02) (table 2).

**Table 2:** Primary and secondary outcomes

	RA group n=25	Infiltration group n=23	P value
**Primary outcome**			
OME at 24 hours	25.5±19.8	45.7±37.6	0.02*
**Secondary outcomes**			
OME			
OME in PACU	12.6±11.6	19.0±14.1	0.08
OME at 48 hours	35.8±30.2	64.5±60.0	0.04*
NRS			
Max NRS in PACU	5 (1.0–6.3)	5 (3.3–7.0)	0.16
Max NRS at 24 hours	5 (3.8–7.0)	6 (4.3–7.8)	0.18
Max NRS at 48 hours	5 (3.0–6.0)	5 (4.3–7.0)	0.48
**Operating room parameters**			
Anesthetic duration (min)	35±14	27±13	0.05
Surgery duration (min)	204±65	209±76	0.78
Surgery blood loss (mL)	800 (588–1163)	700 (463–875)	0.19
**General anesthetic drugs intake**			
Propofol (mg/kg/min)	0.17 (0.15–0.20)	0.17 (0.14–0.19)	0.85
Remifentanil (µg/kg/min)	0.21±0.05	0.22±0.06	0.63
**PONV, n (%)**			
PACU	1 (4)	1 (4)	0.99
0–24 hours	12 (48)	11 (48)	0.99
24–48 hours	8 (32)	10 (44)	0.41

Data are expressed as n (%), mean±SD or median (25th–75th percentiles) as appropriate.

* Significant p value.

NRS, numerical rating scale; OME, oral morphine equivalent; PACU, postanesthesia care unit; PONV, postoperative nausea and/or vomiting; RA, regional anesthesia.

According to ANOVA, the evolution over time of OME consumptions was significantly different between measurements (ie, ‘time effect’, p<0.001), with a significant difference between groups (ie, ‘group effect’, p=0.02). The difference between time measurements depends on group membership (ie, ‘group x time interaction’, p=0.04).

All secondary outcome results are available in table 2. One vascular puncture occurred during a V3 nerve block representing a 1% incidence rate. No local anesthetic systemic toxicity was reported. Interestingly, worst pain score and cumulative OME consumptions on day 2 were positively correlated with the APAIS (Pearson’s correlation coefficients of 0.42 (p<0.01) and 0.39 (p<0.01), respectively).

## Discussion

In this prospective, controlled, randomized, single-blind trial, we demonstrated that a bilateral ultrasound-guided V2 and V3 combined nerves block reduces morphine consumption by 50% in the first 24 postoperative hours in patients undergoing double-jaw orthognathic surgery. Furthermore, there would appear to be no additional risk with this analgesic strategy.

Concerning morphine consumption reduction, the size effect we identified was very close to the ones from previous study who obviously demonstrated an analgesic effect of a V2 block in pediatric cleft palate surgeries.^7^ Our results are also similar with two more recent studies which have demonstrated that V2 or V3 nerve block decreases postoperative opioid intake for maxillary or mandibular osteotomy, respectively.^9 16^ Thus, our study highlights significant advantage of combined nerves block to reduce postoperative pain in patients undergoing double-jaw surgery.

Our results are also close to those reported in two studies investigating the analgesic effect of V2 and V3 combined nerves block in double-jaw surgeries. Indeed, Shetty and colleagues found significant lower postoperative pain scores (NRS at 6, 12, 24 and 48 hours) as well as less analgesic drug consumption in their RA group.^18^ However, opioid consumption was not analyzed because of the socioeconomic conditions of the patients included with no access to opioids. Surprisingly, we found no significant difference in pain scores between groups. First of all, it might be possible that a lack of power brings out significant difference. Moreover, the feeling of pain is not linear and patients remember more the worst pain they experienced (central sensitization) than their pain score at a given, sometimes irrelevant, time point. Finally, our morphine administration protocol is designed to keep pain scores below 3.

Chen and coworkers highlighted a significant but slight decrease in intraoperative fentanyl use during surgery.^17^ In our study, we did not find differences in anesthetic agent consumption between groups. This result could be explained by our standardized anesthetic protocol in which all patients benefited from an intravenous multimodal analgesia including remifentanil, ketamine, paracetamol, non steroidal anti inflammatory drugs (NSAID) and nefopam. Moreover, lidocaine infiltration was systematically performed in patients allocated to the control group. Furthermore, residual pain involving V1 territory uncovered by RA could still remain. Taken together, these parameters can easily explain the similar level of intraoperative analgesia in our two groups of patients. Finally, it is highly probable that the duration of action of lidocaine (about 2 hours with added epinephrine) extends until arrival in the recovery room, explaining the absence of difference in the PACU opioid consumption between groups.^23^ In this setting, peripheral nerve block with ropivacaine (and with the adjunction of intravenous dexamethasone) could explain the reduction in morphine consumption at postoperative days 1 and 2 for patients allocated to the RA group.^24^

In this work, peripheral blocks of the V2 and V3 branches of the trigeminal nerve were performed under ultrasound guidance. We reported one vascular puncture that occurred during a V3 nerve block representing a 1% incidence rate. Thus, we strongly believe that ultrasound guidance could reduce the incidence of vascular punctures.^3^ Several studies evaluating facial nerve block tend to confirm our assumption: the reduction of local anesthetic volume with ultrasound guidance could therefore reduce the associated risks, notably the systemic resorption and toxicity of local anesthetic.^25^ In addition, the V2 suprazygomatic approach is considered safer than the infrazygomatic avoiding meningeal artery puncture, infraorbital or even intracranial fissure injections of local anesthetics.^7 19 26^ It is worth noting that this nerve block is technically identical to the percutaneous suprazygomatic pterygopalatine ganglion block (formerly called sphenopalatine ganglion block) that has already been described and performed for the treatment of acute migraine or postdural puncture headache. Since we believe that block nomenclature should depend on the desired target, we have decided to keep the label maxillary nerve block throughout the study.^27^

Since 1996, the APAIS questionnaire allowed for a better understanding of preoperative anxiety.^28^ Many studies have suggested that the preoperative anxiety was positively correlated to postoperative pain.^29^ The significant correlation between APAIS and postoperative morphine consumption we identified in our study corroborates the existence of this causality. The benefits of identifying anxious patients at risk of intense postoperative pain pave the way for a multidimensional approach of pain for patients scheduled for orthognathic surgery.

Our results must be interpreted with caution and a number of limitations should be borne in mind. First, this study is a single-blind trial. However, only the anesthesia and surgical teams in charge of the patient in the operating room were informed of allocation group. Neither patient nor PACU/outpatient department/surgical ward staff were aware of randomization group to which the subject belongs. Therefore, postoperative opioid administration was carried out only by caregivers who were blinded to randomization. Persons in charge of postoperative data collection and analyses remained blinded to the randomization group. Second, despite the fact we demonstrated that RA exerts an opioid-sparing effect, we did not find any benefit in reducing PONV. Such results have already been described in a recent study evaluating the effectiveness of an ultrasound-guided mandibular nerve block.^16^ Moreover, it has been demonstrated that PONV after orthognathic surgery is the result of a multiple factors interaction including age, gender, pain intensity, surgical duration, use of halogens, consumption of opioids…but is not correlated with anesthetic’s experience.^30^ Further studies are necessary to better understand the risk factor of PONV in double-jaw surgery. Third, for patients in the control group the mucosal surgical infiltration was done with lidocaine, while for patients in the RA group the V2 and V3 nerves block was done with ropivacaine. Further studies comparing ultrasound-guided RA and mucosal surgical infiltration with the same long-acting agent are required before considering RA as the best analgesic option. Finally, in a context of day-case surgery expansion worldwide, cephalic RA seems fully indicated to promote enhanced recovery in maxillofacial surgery, but further studies are required.^31^

## Conclusions

Bilateral ultrasound-guided V2 and V3 combined nerves block is an efficient RA which allows a 50% opioid-sparing effect in the first 24 hours after double-jaw orthognathic surgery. Preoperative anxiety is correlated to postoperative pain and morphine consumption in this setting.

## Supplementary material

10.1136/rapm-2024-105497 online supplemental file 1

10.1136/rapm-2024-105497 online supplemental file 2

## Data Availability

Data are available upon reasonable request.
